# How metaverse music platform cues shape content creation behavior: Evidence from social and flow pathways

**DOI:** 10.1371/journal.pone.0348632

**Published:** 2026-05-04

**Authors:** Qiyue Song, Di Mu, Xiaodi Peng, Guanzhou Liu

**Affiliations:** 1 IQRA Business School, Universiti Geomatika Malaysia, Kuala Lumpur, Malaysia; 2 School of Music and Dance, Heze University, Heze, Shandong, China; 3 The University of Sydney Business School, The University of Sydney, Sydney‌‌, New South Wales, Australia; 4 UCSI Graduate Business School, UCSI University, Kuala Lumpur, Malaysia; Guangdong University of Petrochemical Technology, CHINA

## Abstract

This study examines how metaverse music platform design influences users’ content creation behavior. Drawing on the Stimulus–Organism–Response framework, it develops a dual-path model in which relational-affective cues shape social experience, whereas interaction-oriented cues shape flow experience. Both experiential states are proposed to enhance emotional value perception, which in turn predicts content creation behavior, with technology acceptance examined as a boundary condition. Results from a survey of 435 Chinese users of metaverse music platforms show that emotional resonance and social presence positively predict social experience, whereas aesthetic novelty does not. Avatar customization, narrativity, and multi-sensory positively predict flow experience. Social experience and flow experience both strengthen emotional value perception, which subsequently increases content creation behavior. Technology acceptance strengthens only the social experience route. The study contributes by extending SOR research to metaverse music platforms, clarifying that music-related posting and sharing is shaped through distinct social and flow-based pathways, and showing that technology-related beliefs function as a selective boundary condition rather than a uniform amplifier across the model.

## 1 Introduction

Metaverse music platforms are extending digital music experiences beyond one-way viewing into avatar-mediated, immersive, and interactive virtual environments that support both virtual concert attendance and music-related content creation behavior (CCB) [1,2]. On these platforms, users can attend virtual performances, interact with others in real time, and engage with music-related features through persistent digital identities and in-platform interactive functions [2,3]. Research on platform-based creator ecosystems further suggests that digital platforms do not merely host participation but also structure and support content creation and management [4]. Yet the presence of these features alone does not explain why some users move beyond attendance to post or share music-related content on the platform, whereas others remain primarily attendees.

Existing metaverse research has examined platform attributes, customer experience, engagement, and usage-related outcomes [5–7]. In event- and music-related metaverse research, prior studies have also explored virtual concerts, metaverse events, and avatar-based virtual event experiences [3,8,9]. However, this work has focused mainly on experience quality, authenticity, resistance, satisfaction, and related usage evaluations rather than on users’ music-related posting and sharing behavior on these platforms [5,10]. As a result, less is known about how platform cues are translated through experiential and evaluative processes into CCB in this platform context.

To address this gap, the present study adopts the Stimulus–Organism–Response (SOR) framework [11,12]. It develops a dual-path model in which relational-affective cues are expected to strengthen social experience (SE), whereas interaction-oriented cues are expected to strengthen flow experience (FE). These experiential states are then proposed to enhance emotional value perception (EVP), which in turn predicts CCB. Technology acceptance (TA) is further included as a boundary condition that may shape how experience is translated into EVP [13].

This study examines the proposed mechanism among Chinese users of metaverse music platforms. This empirical focus is appropriate because immersive entertainment, platform-based social interaction, and digital content practices are especially prominent in chinese metaverse music platform context [3,14,15]. Rather than treating China as a separate theoretical issue, the study uses this context to provide a clearer view of that process. Against this background, the study investigates how platform cues are translated into CCB.

This study contributes to research on metaverse platforms by using the SOR framework to explain a downstream response in a metaverse music-oriented platform context. First, it positions CCB as the focal response outcome in this domain. Second, it explains how two broad forms of platform design, relational-affective cues and interaction-oriented cues, are linked to that response through social experience, flow experience, and emotional value perception. Third, it examines whether technology acceptance conditions the translation of experience into emotional value. In doing so, the study extends prior SOR-based work on virtual concerts, VR, and related immersive digital settings by offering a more focused explanation in the specific context of Chinese users of such platforms.

## 2 Theoretical framework and proposed hypotheses

### 2.1 Stimulus–Organism–Response (SOR)

Mehrabian and Russell proposed the Stimulus–Organism–Response (SOR) framework to explain how controllable external stimuli shape internal organismic states and subsequently elicit behavioral responses [16]. In the context of metaverse music platforms, this framework is useful because it distinguishes among what platforms design, what users experience, and what users ultimately do. This distinction is especially relevant in metaverse music platforms, where users participate in avatar-mediated and interactive environments rather than merely consume content. Building on prior applications of SOR in digital experience research, the present study uses this framework to explain how platform design cues shape user experience states and, through value appraisal, motivate CCB [17,18].

In the proposed model, platform design cues function as stimuli; SE and FE are modeled as first-stage organismic states; EVP is modeled as a subsequent evaluative appraisal; and CCB is the focal response. At the stimulus level, the model distinguishes between two groups of design cues. The first comprises relational-affective cues: emotional resonance (ER), aesthetic novelty (AN), and social presence (SP), which are expected to primarily strengthen SE. The second comprises interaction-oriented cues: avatar customization (AC), narrativity (NA), and multi-sensory (MS), which are expected to primarily strengthen FE. This structure makes it possible to distinguish how different forms of platform design are associated with different experience states before these experiences are translated into value perception and subsequent posting or sharing behavior.

**Fig 1:**
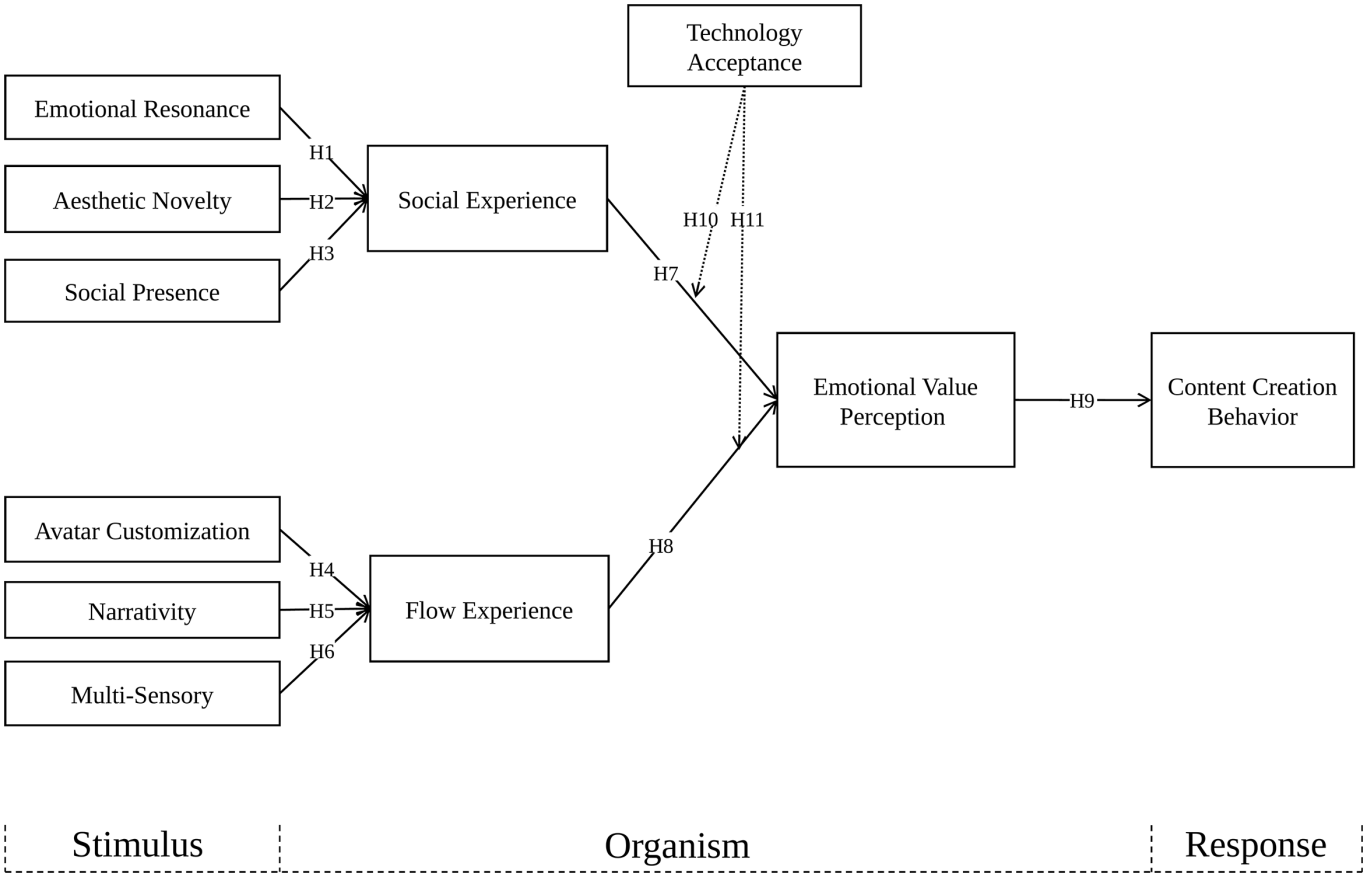
Conceptual research model.

A single undifferentiated organism layer is not sufficient in the present study because CCB on metaverse music platforms represents a post-attendance content behavior that is often visible to others [4,19]. Users may enjoy a platform encounter without necessarily moving beyond attendance into this downstream response. Accordingly, the organism layer is modeled as two parallel first-stage states followed by a subsequent appraisal stage. SE reflects the relational quality of participation, FE reflects absorbed involvement and perceived control, and EVP captures whether these experiences are appraised as emotionally valuable enough to support that downstream response. Fig 1 presents the proposed conceptual model.

### 2.2 Relational-affective cues and social experience

Metaverse music platforms offer more than technical interaction alone. They also present relational and affective cues that shape how participation is experienced socially. In the present study, ER, AN, and SP are selected as relational-affective cues because they can be directly perceived during participation, can be strengthened through platform and performance design, and are more proximally related to SE than to FE. Although these cues may co-occur during the same platform episode, each reflects a distinct social condition through which participation comes to feel shared and meaningful.

ER refers to users’ perceived emotional alignment with a performance and its interactive framing. It reflects whether the emotional tone of the music-related experience feels expressive, meaningful, and shareable within the platform environment [20]. AN refers to users’ perception of originality, surprise, and creative distinctiveness in audiovisual presentation [21]. Although ER and AN may coexist, they are conceptually distinct. ER provides an affective basis for connection, whereas AN introduces distinctiveness that can attract curiosity and shared attention. SP, in turn, refers to users’ perception that others are present in the shared virtual environment [22]. It is reflected in cues such as the visibility of other participants, synchronous communication, and awareness of a shared space. By contrast, SE refers to the relational experience state that emerges when participation becomes socially meaningful. It reflects whether participation feels connecting, supportive, inclusive, and collectively engaging [20,23]. Thus, SP captures the perception that others are present, whereas SE captures the experienced quality of social participation once interaction unfolds.

These cues contribute to SE through related but distinct mechanisms. ER can make participation feel more emotionally shared, thereby supporting stronger audience connection and engagement [24]. AN can draw collective attention to the experience and encourage conversation around it, thereby strengthening the sense of co-experience [25]. SP provides the basic sense of co-presence from which connection, inclusion, and collective involvement can develop [23]. Accordingly, the following hypotheses are proposed:

*H1: Emotional resonance positively influences social experience.*

*H2: Aesthetic novelty positively influences social experience.*

*H3: Social presence positively influences social experience.*

### 2.3 Interaction-oriented cues and flow experience

Metaverse music platforms also rely on design cues that support attention, agency, and sustained involvement. In the present study, AC, NA, and MS are treated as interaction-oriented cues because they are directly experienced during participation and are more closely related to acting, navigating, and remaining engaged than to relational interpretation. Although these cues may also arise within the same platform encounter, they represent distinct interactional conditions that help sustain attention, control, and immersive involvement.

AC refers to users’ ability to shape self-presentation and identity expression through their avatars [26]. By allowing users to influence how they appear and act in the virtual environment, AC can strengthen perceived agency and personal involvement [27]. NA refers to coherent storyline cues and goal-relevant progression that organize participation into meaningful episodes [28]. It provides direction and continuity, helping users remain oriented as they move through platform-based sequences and interactions. MS refers to sensory-enriching inputs, such as spatial audio and embodied or haptic feedback, that deepen perceptual engagement and help sustain attention during participation [29,30].

FE is an optimal experience state characterized by deep absorption, focused attention, perceived control, and intrinsic enjoyment [31]. It includes immersion as an experiential component, but it is not reducible to immersion alone. In the present study, FE is defined by the joint presence of absorption and perceived control rather than by sensory vividness alone. This distinction matters because music-related posting and sharing require more than momentary immersion; they also depend on stable agency and attentional continuity during participation. From this perspective, AC is expected to strengthen FE by enhancing control and involvement, NA by sustaining direction and reducing cognitive drift, and MS by anchoring attention through richer sensory input. Accordingly, the following hypotheses are proposed:

*H4: Avatar customization positively influences flow experience.*

*H5: Narrativity positively influences flow experience.*

*H6: Multi-sensory positively influences flow experience.*

### 2.4 Emotional value perception

EVP refers to users’ appraisal of a metaverse music experience as emotionally rewarding and meaningful [10,32]. In the present study, EVP captures whether SE and FE are appraised positively enough to motivate post-attendance expression. When the experience is perceived as emotionally valuable, users should be more likely to post or share music-related content on the platform. SE is expected to strengthen EVP because shared and connected participation can increase the emotional value users attach to the experience, whereas FE is expected to strengthen EVP because focused and self-directed participation can make the experience feel more worthwhile. Accordingly, the following hypotheses are proposed:

*H7: Social experience positively influences emotional value perception.*

*H8: Flow experience positively influences emotional value perception.*

### 2.5 Content creation behavior

CCB is the focal response outcome in this study because it captures whether users extend their participation beyond attendance into visible platform expression. In the present study, CCB refers specifically to users’ posting or sharing of music-related textual or visual content on metaverse music platforms, such as posts or articles, status updates, and photos or videos related to their participation [33]. As operationalized here, CCB does not include liking, chatting, browsing, or other low-effort reactions to existing content. Compared with more commonly examined outcomes such as engagement or continued use, this construct captures a more specific form of music-related posting and sharing on the platform [5,6]. Because such behavior requires users to continue expressing their participation after the focal experience, stronger EVP should increase their willingness to invest effort in posting or sharing music-related content [4,19]. Accordingly, the following hypothesis is proposed:

*H9: Emotional value perception positively influences content creation behavior.*

### 2.6 Technology acceptance

TA reflects users’ judgments about a platform’s usefulness and ease of use and is commonly used to explain adoption and continued engagement [13,34]. In metaverse music platforms, such beliefs can influence how smoothly users interpret and continue participation. Such judgments become especially important when experience is translated into EVP. When TA is high, interface clarity and perceived system adequacy reduce friction, making it more likely that SE and FE will be appraised as emotionally rewarding and meaningful. When TA is low, perceived difficulty and system-related disruptions can weaken this appraisal process [13]. In the present model, design stimuli capture episode-level cues during participation, whereas TA reflects a more stable platform-level belief formed across repeated interactions. EVP remains the most proximal value appraisal explaining CCB, with TA conditioning how experience states consolidate into that appraisal. Accordingly, the following hypotheses are proposed:

*H10: Technology acceptance moderates the relationship between social experience and emotional value perception.*

*H11: Technology acceptance moderates the relationship between flow experience and emotional value perception.*

## 3 Methods

### 3.1 Study context, sampling, and data collection

This study employed a cross-sectional, self-administered online survey to test the proposed model [35]. Eligibility was verified through the screening questions reported in S1 Appendix. Specifically, respondents had to be at least 18 years old, have used a metaverse music platform in the past 12 months, and report prior experience with avatar-based participation, in-platform interactive features, and at least one relevant platform activity. The questionnaire also included background items on platform use to describe respondents’ familiarity with the focal context.

The survey was administered online via Sojump using purposive sampling [36]. Participants were recruited through Chinese online communities linked to social VR worlds, creator-centered avatar platforms, and game-based virtual music environments. To establish the empirical context, respondents were asked to indicate which metaverse music platforms they had used for avatar-based, music-related, and interactive virtual participation. The most frequently reported platforms were Roblox (33.8%), Fortnite (31.0%), and VRChat (28.3%). Of the 500 questionnaires initially collected, 435 valid responses were retained after incomplete and ineligible cases were removed. The questionnaire was anonymous, collected no direct identifiers, obtained informed e-consent before participation, and offered no incentives. The final sample therefore comprised qualified users with relevant metaverse music platform experience. Table 1 reports the demographic profile and platform-use characteristics of the sample, including age, gender, educational level, use frequency, and duration of use.

**Table 1:** Sample characteristics (n = 435).

Characteristics	Specifications	Frequency	Percentage
Age	18-29 years old	183	42.07
	30-43 years old	121	27.82
	44-59 years old	89	20.46
	60 years and above	42	9.66
Gender	Male	243	55.86
	Female	192	44.14
Educational Level	High school	91	20.92
	Bachelor’s degree	214	49.20
	Master’s degree	89	20.46
	Doctoral degree	41	9.43
Frequency of Metaverse Music Platform Usage	Only once	83	19.08
	2-5 times	135	31.03
	6-10 times	101	23.22
	11-20 times	68	15.63
	21 times or more	48	11.03
Duration of Metaverse Music Platform Usage	Less than 3 months	133	30.57
	3-6 months	95	21.84
	6 months – 1 year	127	29.20
	More than 1 year	80	18.39
Total		435	100.0

### 3.2 Measures development

For the substantive constructs, all items were measured on five-point Likert scales ranging from 1 = strongly disagree to 5 = strongly agree. To keep the instrument manageable in a multi-construct model and reduce respondent burden, three representative items were retained for each focal construct. Item selection was guided by conceptual correspondence with the construct definitions, contextual relevance to metaverse music platforms, and prior evidence of sound psychometric performance. This parsimonious strategy reduced respondent burden, although it may have captured less construct breadth than longer scales. The questionnaire was initially developed in English and then translated into Chinese using Brislin’s forward-backward translation procedure [37]. After minor wording refinements, the instrument was pilot-tested with 30 participants to assess clarity, readability, and contextual appropriateness; these pilot responses were excluded from the final sample.

Specifically, emotional resonance (ER) was adapted from Gu [38], aesthetic novelty (AN) from Blomstervik et al. [21], social presence (SP) from Oh et al. [23], social experience (SE) from Onderdijk et al. [39], avatar customization (AC) from Lee et al. [40], narrativity (NA) from Bilandzic et al. [41], multi-sensory (MS) from Gómez-Suárez and Yagüe [42], flow experience (FE) from Norsworthy et al. [43], emotional value perception (EVP) from Choi et al. [10], technology acceptance (TA) from Al-Adwan et al. [34], and content creation behavior (CCB) from Chen et al. [33].

To improve measurement transparency, several example items are provided here. For instance, SP included the item “I had a sense that I was interacting with other people in this experience, not just with the system.” FE included “During this metaverse music experience, I was absorbed in what I was doing,” and CCB included “I post photos/videos on metaverse music platforms.” The full set of measurement items and sources is reported in S2 Appendix.

### 3.3 Analytical strategy and bias control

The analyses were conducted using SPSS 28 and AMOS 23. Using maximum likelihood estimation, we first assessed the measurement model through confirmatory factor analysis (CFA) and then estimated the structural model to test the hypothesized direct effects and serial indirect relationships [44]. Measurement quality was evaluated using standardized factor loadings, Cronbach’s alpha [45], composite reliability (CR), average variance extracted (AVE), and discriminant validity [46]. Model fit was assessed using χ², df, χ²/df, CFI, TLI, and RMSEA, and indirect effects were examined using 5,000 bootstrapped confidence intervals [47,48].

To test the moderating role of TA, we conducted a supplementary hierarchical regression analysis based on composite scores. SE, FE, and TA were mean-centered prior to analysis, and the interaction terms (SE × TA and FE × TA) were computed from these centered variables. This regression-based analysis was used to test the moderation hypotheses outside the latent structural model, whereas the main SEM was retained to estimate the serial mediation structure [49]. Potential common method bias (CMB) was assessed using Harman’s single-factor test and a common latent factor (CLF) approach [50].

## 4 Results

### 4.1 Measurement model assessment

Before testing the measurement model, potential CMB was assessed. Harman’s single-factor test showed that the first unrotated factor explained 22.05% of the total variance, below the 50% threshold [51]. A CLF test further indicated only limited differences between the baseline CFA model and the CLF-adjusted model (χ² = 484.32, CFI = 0.992, TLI = 0.990, RMSEA = 0.021), with an average absolute change in standardized loadings of 0.060. These results suggest that CMB was not a serious concern.

The measurement model was evaluated using CFA. The CFA showed good fit (χ² = 569.702, df = 440, χ²/df = 1.295, CFI = 0.987, TLI = 0.984, RMSEA = 0.026). As shown in Table 2, all standardized factor loadings were significant and ranged from 0.720 to 0.926. Cronbach’s alpha values ranged from 0.851 to 0.907, CR values ranged from 0.861 to 0.918, and AVE values ranged from 0.679 to 0.790. These results indicate satisfactory reliability and convergent validity [52].

**Table 2:** Construct reliability and validity.

Construct	Items	Loadings	Cronbach’s alpha	CR	AVE
Emotional Resonance [38]	ER1	0.905	0.892	0.899	0.751
	ER2	0.780			
	ER3	0.800			
Aesthetic Novelty [21]	AN1	0.873	0.851	0.861	0.679
	AN2	0.720			
	AN3	0.749			
Social Presence [23]	SP1	0.908	0.888	0.898	0.749
	SP2	0.820			
	SP3	0.785			
Social Experience [39]	SE1	0.894	0.892	0.900	0.751
	SE2	0.799			
	SE3	0.804			
Avatar Customization [40]	AC1	0.897	0.886	0.896	0.743
	AC2	0.786			
	AC3	0.807			
Narrativity [41]	NA1	0.926	0.907	0.918	0.790
	NA2	0.815			
	NA3	0.823			
Multi-Sensory [42]	MS1	0.890	0.884	0.891	0.733
	MS2	0.788			
	MS3	0.796			
Flow Experience [43]	FE1	0.899	0.883	0.892	0.737
	FE2	0.785			
	FE3	0.787			
Emotional Value Perception [10]	EVP1	0.912	0.893	0.902	0.757
	EVP2	0.804			
	EVP3	0.782			
Technology Acceptance [34]	TA1	0.871	0.886	0.886	0.723
	TA2	0.791			
	TA3	0.806			
Content Creation Behavior [33]	CCB1	0.922	0.906	0.916	0.786
	CCB2	0.811			
	CCB3	0.837			

Table 3 reports the means, standard deviations, inter-construct correlations, and Fornell–Larcker assessment. The square roots of AVE exceeded the corresponding inter-construct correlations for all constructs, supporting discriminant validity. In addition, variance inflation factors (VIFs) ranged from 1.033 to 1.327, indicating no evident multicollinearity concern [53]. Correlations involving TA, and to a lesser extent AN, were weak and occasionally negative, which is consistent with their more peripheral role in the main experiential chain relative to the focal stimulus-to-state links. In this model, several effects are indirect or conditional, so small zero-order correlations do not preclude meaningful net effects once mediators and competing predictors are estimated simultaneously. Overall, these results support the adequacy of the measurement model and provide a suitable basis for the subsequent structural analysis.

**Table 3:** Descriptive statistics, correlations, and discriminant validity.

Construct	Mean	SD	ER	AN	SP	SE	AC	NA	MS	FE	EVP	TA	CCB
ER	3.726	0.747	**0.866**	−0.020	0.058	0.194	0.215	0.077	0.198	0.230	0.301	−0.063	0.170
AN	3.881	0.644	−0.020	**0.824**	−0.087	0.051	−0.073	0.080	−0.004	−0.014	−0.033	0.009	0.007
SP	3.572	0.785	0.058	−0.087	**0.865**	0.279	0.281	0.270	0.297	0.334	0.212	−0.085	0.216
SE	3.657	0.772	0.194	0.051	0.279	**0.867**	0.265	0.275	0.333	0.310	0.245	−0.173	0.148
AC	3.697	0.764	0.215	−0.073	0.281	0.265	**0.862**	0.232	0.290	0.285	0.241	−0.075	0.216
NA	3.533	0.884	0.077	0.080	0.270	0.275	0.232	**0.889**	0.313	0.289	0.239	−0.088	0.107
MS	3.792	0.699	0.198	−0.004	0.297	0.333	0.290	0.313	**0.856**	0.307	0.241	−0.053	0.224
FE	3.679	0.759	0.230	−0.014	0.334	0.310	0.285	0.289	0.307	**0.858**	0.265	−0.078	0.134
EVP	3.695	0.771	0.301	−0.033	0.212	0.245	0.241	0.239	0.241	0.265	**0.870**	−0.057	0.132
TA	3.642	1.116	−0.063	0.009	−0.085	−0.173	−0.075	−0.088	−0.053	−0.078	−0.057	**0.851**	−0.054
CCB	3.415	0.898	0.170	0.007	0.216	0.148	0.216	0.107	0.224	0.134	0.132	−0.054	**0.887**

Diagonal elements in bold are the square roots of average variance extracted (AVE); off-diagonal elements are inter-construct correlations.

### 4.2 Structural model results and hypothesis testing

The structural model showed good fit (χ² = 747.633, df = 465, χ²/df = 1.608, CFI = 0.972, TLI = 0.968, RMSEA = 0.037). The direct path estimates are reported in Table 4. Overall, eight of the nine direct hypotheses were supported. The model explained 16.4% of the variance in SE, 20.3% in FE, 10.0% in EVP, and 2.6% in CCB. This pattern indicates that the proposed model explains intermediate experiential states more strongly than the final behavioral outcome. Although EVP significantly predicted CCB, the explained variance for CCB remained low (R² = 0.026), suggesting that the proposed mechanism captures one meaningful route to music-related posting and sharing behavior rather than providing a complete explanation of that behavior.

**Table 4:** Structural path estimates and hypothesis testing.

Hypothesis	Path	Std. β	B	SE	CR	p	Decision
H1	ER → SE	0.184	0.194	0.048	4.026	< 0.001	Supported
H2	AN → SE	0.060	0.072	0.055	1.327	0.184	Not supported
H3	SP → SE	0.343	0.326	0.044	7.467	< 0.001	Supported
H4	AC → FE	0.226	0.224	0.047	4.807	< 0.001	Supported
H5	NA → FE	0.188	0.164	0.040	4.079	< 0.001	Supported
H6	MS → FE	0.203	0.226	0.053	4.229	< 0.001	Supported
H7	SE → EVP	0.185	0.181	0.046	3.951	< 0.001	Supported
H8	FE → EVP	0.240	0.235	0.045	5.205	< 0.001	Supported
H9	EVP → CCB	0.161	0.192	0.057	3.379	< 0.001	Supported

Std. β = standardized coefficient; B = unstandardized coefficient; SE = standard error; CR = critical ratio.

On the relational-affective pathway, ER (β = 0.184, p < 0.001) and SP (β = 0.343, p < 0.001) positively predicted SE, supporting H1 and H3, whereas AN did not significantly predict SE (β = 0.060, p = 0.184); thus, H2 was not supported. On the interaction-oriented pathway, AC (β = 0.226, p < 0.001), NA (β = 0.188, p < 0.001), and MS (β = 0.203, p < 0.001) all positively predicted FE, supporting H4–H6. At the subsequent stages of the model, both SE (β = 0.185, p < 0.001) and FE (β = 0.240, p < 0.001) positively predicted EVP, supporting H7 and H8. EVP, in turn, positively predicted CCB (β = 0.161, p < 0.001), supporting H9. These results are summarized in Table 4.

### 4.3 Serial indirect effects analysis

To further assess the proposed process logic, we tested the serial indirect effects from the six stimulus variables to CCB through the corresponding experience state and EVP. The results are reported in Table 5. On the SE pathway, ER and SP showed significant serial indirect effects on CCB through SE and EVP, whereas the serial indirect effect of AN was not significant. On the FE pathway, AC, NA, and MS all showed significant serial indirect effects on CCB through FE and EVP. These results are consistent with the proposed staged mechanism in which metaverse music platform cues relate to CCB through experience formation and emotional value appraisal.

**Table 5:** Serial indirect effects on CCB.

Serial indirect path	Indirect effect	Bootstrap SE	95% bootstrap CI
ER → SE → EVP → CCB	0.006	0.004	[0.001, 0.016]
AN → SE → EVP → CCB	0.002	0.002	[-0.011, 0.007]
SP → SE → EVP → CCB	0.011	0.006	[0.002, 0.025]
AC → FE → EVP → CCB	0.009	0.005	[0.001, 0.020]
NA → FE → EVP → CCB	0.007	0.004	[0.001, 0.017]
MS → FE → EVP → CCB	0.008	0.005	[0.001, 0.019]

Indirect effects are considered significant when the 95% bootstrap confidence interval does not include zero.

### 4.4 Moderation effects of technology acceptance

**Fig 2:**
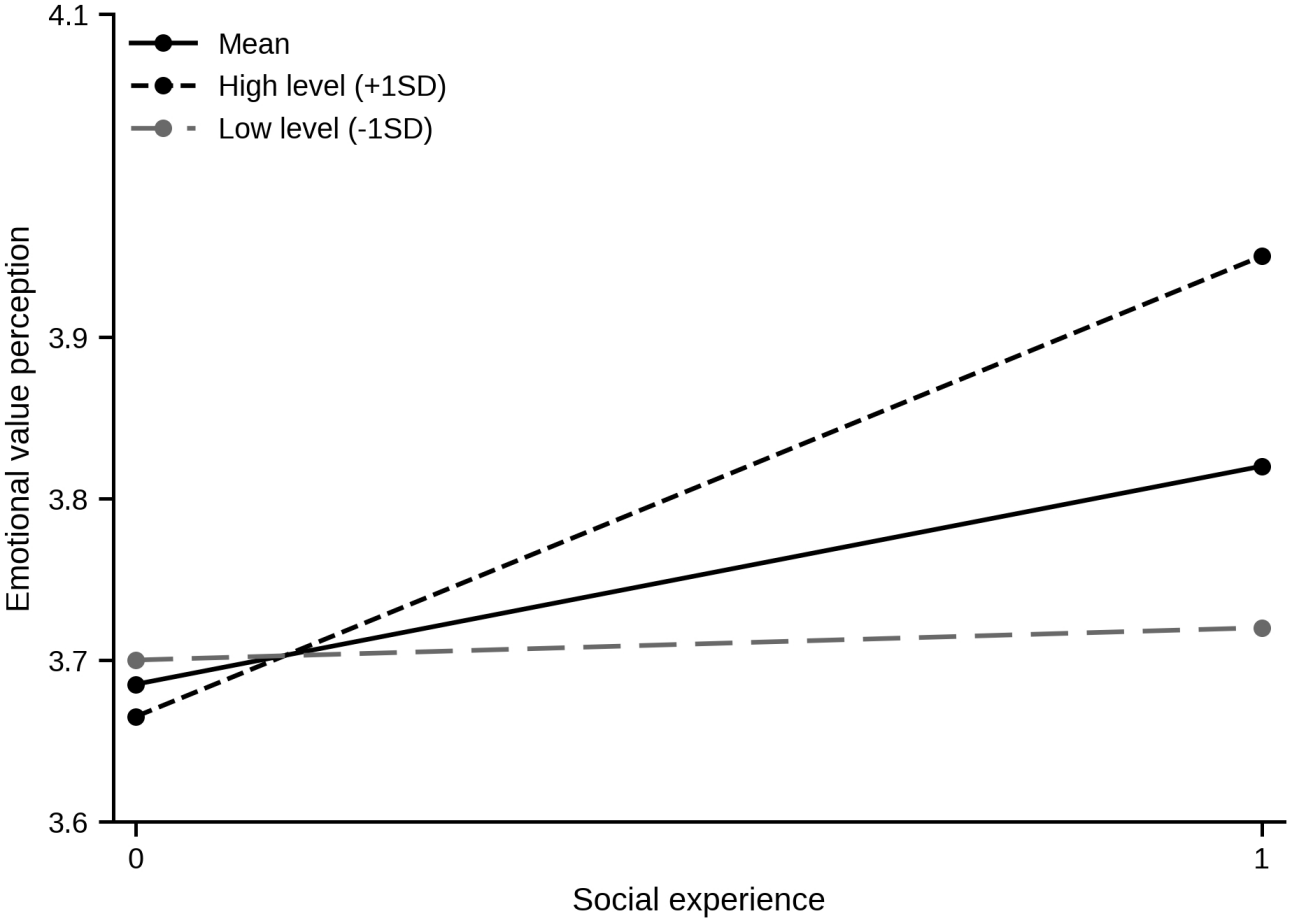
Interaction effect of social experience and technology acceptance on emotional value perception.

As a supplementary boundary-condition test, we examined whether TA moderates the effects of SE and FE on EVP using hierarchical regression. Before estimating the moderation models, SE, FE, and TA were mean-centered, and the interaction terms were then computed from these centered variables. The results are reported in Table 6. The SE × TA interaction was significant (B = 0.113, p = 0.011), supporting H10. Adding the interaction term increased the explained variance in EVP from R^2^ = 0.060 to R^2^ = 0.075 (ΔR^2^ = 0.015). As shown in Fig 2, the positive association between SE and EVP was stronger at higher levels of TA. By contrast, the FE × TA interaction was not significant (B = 0.063, p = 0.168), so H11 was not supported. These results indicate that TA conditions the SE → EVP relationship, whereas the FE → EVP relationship remains relatively stable across levels of TA. Although the incremental explanatory power was modest, this finding suggests that higher TA can strengthen the emotional value users derive from socially shared experiences.

**Table 6:** Moderation effects of technology acceptance.

Panel A. SE × TA → EVP												
	Model 1				Model 2				Model 3			
Predictor	B	SE	t	p	B	SE	t	p	B	SE	t	p
SE	0.245	0.047	5.268	< 0.001	0.242	0.047	5.127	< 0.001	0.224	0.048	4.716	< 0.001
TA					−0.010	0.033	−0.319	0.750	−0.025	0.033	−0.747	0.455
SE × TA									0.113	0.044	2.566	0.011
R²	0.060				0.060				0.075			
ΔR²	—				0.000				0.015			
**Panel B. FE × TA → EVP**												
FE	0.269	0.047	5.709	< 0.001	0.266	0.047	5.627	< 0.001	0.253	0.048	5.256	< 0.001
TA					−0.025	0.032	−0.791	0.429	−0.031	0.032	−0.957	0.339
FE × TA									0.063	0.046	1.382	0.168
R²	0.070				0.071				0.075			
ΔR²	—				0.001				0.004			

Predictors were mean-centered before creating the interaction terms. Reported p values are two-tailed.

## 5 Discussion

### 5.1 Main pathways to content creation behavior

One clear pattern in the findings is that social experience was shaped mainly by cues related to social presence and emotional resonance. Social presence appears to strengthen social experience because it reduces the sense of participating alone and makes the event feel shared in real time. When other users seem present and responsive, participation is easier to interpret as a collective event rather than as isolated media consumption. Emotional resonance adds a further layer by aligning users affectively with the performance and with others around it. This affective alignment can turn simple co-presence into a stronger sense of togetherness.

In the Chinese metaverse music context examined here, users are familiar with platform environments in which interaction is highly visible and socially responsive features are expected, especially in avatar-based metaverse platforms where stronger social presence significantly predicts more supportive interaction among users [23]. In this setting, social presence and emotional resonance may matter more because they make participation feel socially shared rather than merely technically enabled. This pattern may also reflect broader platform culture, as many metaverse music experiences take place in game-like ecosystems where avatar co-presence, synchronous interaction, and rapid audience feedback are common [14]. Under such conditions, users may be more sensitive to cues that strengthen the sense of coordinated and collective participation.

Aesthetic novelty, by contrast, did not strengthen social experience. This is a useful boundary finding rather than a failed result. Novel presentation can attract attention, but social experience depends more on whether users feel included, connected, and part of a shared moment [54]. In platform environments where visually rich presentation is already common, novelty may draw attention without necessarily deepening social experience. For experienced Chinese users of metaverse music platforms, visual distinctiveness may therefore be treated as part of the expected environment rather than as a cue that intensifies social participation. Put differently, being striking is not the same as being socially meaningful.

A second pattern concerns the flow route, which was more responsive to cues that helped users remain engaged while maintaining direction and control during participation. Avatar customization may strengthen flow by allowing users to manage how they appear and act on the platform, thereby making participation feel more self-directed. Narrativity can support flow by providing a coherent internal sequence that users can follow, which helps sustain involvement and reduces fragmentation. Multi-sensory may also reinforce flow because it keeps attention anchored in the unfolding activity and makes disengagement less likely. Taken together, these cues seem to support flow not simply by making the platform more vivid, but by making participation easier to sustain, follow, and control over time [12].

What connects both routes to content creation behavior is not the experience state alone, but whether users ultimately regard the experience as emotionally worthwhile enough to extend their involvement beyond attendance [10]. Social experience may be more strongly associated with this response when participation carries interpersonal meaning and value. Flow experience, by contrast, may matter more when focused involvement makes the activity feel personally rewarding and worth continuing beyond the event itself. This pattern helps explain why emotional value perception is positioned so close to the focal response in the model.

### 5.2 Technology acceptance as a boundary condition

Technology acceptance appears to matter more on the social side because socially shared participation is easier to weaken. Feeling connected to others is not enough when the platform still feels awkward or difficult to use, since social moments depend on a basic sense that interaction can unfold smoothly and without interruption [13]. This aspect may be especially important in the present Chinese context, where users are accustomed to socially active platform environments and may therefore be more sensitive to whether shared participation feels smooth or obstructed. In such platform cultures, smooth group interaction is often treated as a basic expectation rather than an added benefit. When that expectation is met, social experience is more likely to be interpreted as emotionally valuable; when it is not, the same moment may remain pleasant but fail to carry enough value to support further commitment.

The flow route appears different. Once users become deeply involved in an activity they can follow and control, its emotional value seems to stem more from the activity itself than from general beliefs about the platform. In that case, platform acceptance still matters in a broad sense, but it does not appear to shape the value of the experience as directly as it does on the social side. This difference helps explain why flow experience appears less dependent on technology acceptance in the present model. The two routes therefore differ not only in what strengthens them but also in how easily their value can be weakened by platform-level judgments.

## 6 Implications

### 6.1 Theoretical implications

This study advances metaverse music platform research by demonstrating that two distinct experiential routes can explain music-related posting and sharing. Relational-affective cues primarily shaped social experience, whereas interaction-oriented cues primarily shaped flow experience. This extends prior SOR-based work in virtual concerts, VR, and related immersive settings by focusing on a more specific response outcome [1,6].

A second theoretical implication is that not all platform design cues contribute equally to this downstream response. In particular, aesthetic novelty should not be treated as a sufficient basis for socially meaningful participation. This sharpens metaverse research by showing that perceptual distinctiveness alone does not create the relational quality needed for content-related expression. The value of this finding therefore lies not simply in reporting a non-significant path, but in clarifying what aesthetic novelty can and cannot explain in socially oriented platform participation.

A third theoretical implication concerns the role of technology acceptance. Rather than acting as a general amplifier of experiential effects, technology acceptance is better understood as a selective boundary condition. In this model, it matters most when socially shared participation is converted into emotional value perception. This shows that beliefs about usability and ease of use are especially important for the value derived from social experience [13]. The implication here is one of conceptual precision: technology-related beliefs do not shape all experiential routes in the same way, and their role is better understood as pathway-specific rather than uniform across the model.

A final theoretical implication is that the Chinese context examined here makes the transition from attendance to platform expression especially visible. These socially responsive, interaction-rich environments help reveal how platform design cues encourage users to move beyond attendance and engage in content creation. In this way, the study also clarifies a pathway logic that may inform theorizing in other immersive digital settings by highlighting how platform design can support post-attendance expression.

### 6.2 Practical implications

Managerially, social and community teams should focus on strengthening the relational quality of participation. The central task is to make users feel acknowledged, connected, and part of a shared moment rather than merely co-located in the same virtual environment. Social presence and emotional resonance should therefore be treated as key levers for strengthening social experience because they enhance mutual awareness, responsiveness, and shared attention [11,23]. Community formats, interaction rituals, and event structures should be designed to make participation feel socially meaningful enough to encourage users to express their participation beyond the event itself.

At the product and UX level, the key issue is whether this downstream response feels straightforward, continuous, and easy to manage. Avatar customization may still matter because it strengthens agency and self-expression during participation [26,27]. Narrativity and multi-sensory features are also useful when they support continuity and attentional anchoring rather than distraction [12]. In practical terms, this implies interfaces that minimize interruption, controls that remain understandable, and content-related functions that make platform expression easier to complete.

A third priority concerns onboarding and interface usability. Because technology acceptance strengthened the social route, platforms should pay particular attention to interface clarity, intuitive navigation, and reliable communication support during early participation [13]. These features are important not only for usability in a narrow technical sense, but also for helping socially shared experiences retain enough value to encourage posting and sharing behavior.

## 7 Limitations and future research

This study has several limitations that also point to directions for future research. First, the study is based on a single-wave, self-reported survey, which limits temporal inference and precludes strong causal claims [55]. In addition, the focal constructs were measured with short scales, and content creation behavior was captured through self-reports rather than through behavioral trace data. Future research could therefore use longitudinal, experimental, diary-based, or platform-log designs to examine whether the proposed pathways remain stable over time and whether reported content creation behavior corresponds to actual platform behavior [56].

Second, the present findings are situated within a specific empirical context. The sample consisted of Chinese users of metaverse music platforms and was recruited through online communities associated with relevant platform ecosystems. This context may shape how users experience and respond to such environments, and the generalizability of the findings may therefore differ across countries, user segments, and platform types. Prior research has shown that the key drivers of online social behavior differ substantially across cultural environments, with utilitarian value and social influence being more salient in some contexts and relational factors being more salient in others [57]. Future research could usefully test the model across different cultural and platform contexts.

Third, the model is deliberately parsimonious, and the moderating effects were tested using a complementary regression-based interaction approach rather than being estimated directly within the latent SEM framework. Although this provides an interpretable test of boundary conditions, it also separates the moderation analysis from the main latent model. Future research could estimate latent interaction models within SEM to examine these interactions within a fully integrated latent-variable framework and compare the results with multigroup or multilevel approaches [58].

## Supporting information

S1 Appendix Complete questionnaire. (DOCX)

S2 Appendix Measurement items. (DOCX)
