# Supplementary material for: How metaverse music platform cues shape content creation behavior: Evidence from social and flow pathways
Source: PLoS One. 2026 May 4;21(5):e0348632. doi: 10.1371/journal.pone.0348632 (PMC13138650; doi:10.1371/journal.pone.0348632)
Supplement: S1 Appendix — (DOCX) [file pone.0348632.s001.docx]

**S1 Appendix**

**Complete Questionnaire**

*English translation of the administered questionnaire*

**Participant Information and Consent**

Welcome to the study!

This survey is part of an academic study on metaverse music platforms. Your participation is voluntary. The survey is anonymous and collects no direct identifiers (e.g., name, phone number, ID). Your answers will be used for research purposes only and reported in aggregate. By selecting ‘I agree’ and proceeding, you confirm that you are at least 18 years old and provide informed e-consent.

By clicking "I agree" and proceeding to the questionnaire, you confirm that you are at least 18 years old and that you consent to participate.

□ Yes, I agree (continue)

□ No (end survey)

**Scenario and key definition**

For this survey, a “metaverse music platform” refers to an avatar-mediated, immersive, and interactive virtual environment where users can attend virtual concerts and engage in music-related participation and expression, including social interaction and music-related posting or sharing.

Response format

Unless otherwise stated, please rate each statement on a 5-point Likert scale: 1 = Strongly disagree, 2 = Disagree, 3 = Neutral, 4 = Agree, 5 = Strongly agree.

**Section A. Screening questions**

The following questions determine whether you are eligible to participate. If you do not meet the eligibility criteria, the survey will end automatically and the termination message will be shown.

Q1. Age group

□ Under 18 (end survey)

□ 18–29 years old

□ 30–43 years old

□ 44–59 years old

□ 60 years and above

Q2. In the past 12 months, have you used a metaverse music platform (as defined above)?

□ Yes

□ No (end survey)

Q3. In the past 12 months, did your use involve avatar-based participation?

□ Yes

□ No (end survey)

Q4. In the past 12 months, did your use involve in-platform interactive or creation tools?

□ Yes

□ No (end survey)

Q5. In the past 12 months, which of the following have you done on a metaverse music platform?

□ Attended at least one metaverse concert/event

□ Created at least one music-related content output (e.g., posting, sharing, uploading)

□ I did not attend any metaverse concert/event and did not create any music-related content (end survey)

**Section B. Platform use background**

Q6. Frequency of general metaverse use (past 12 months)

□ Less than once a month

□ 1–3 times per month

□ 1–2 times per week

□ 3+ times per week

Q7. Frequency of metaverse music platform use (past 12 months)

□ Once only

□ 2–5 times

□ 6–10 times

□ 11–20 times

□ 21 times or more

Q8. Duration of metaverse music platform use

□ Less than 3 months

□ 3–6 months

□ 6 months–1 year

□ More than 1 year

Q9. Which of the following metaverse music platforms have you used for music-related activities in the past 12 months?

□ Roblox

□ VRChat

□ Fortnite

□ Other, please specify: ______

**Section C. Demographic information**

Q10. Gender

□ Male

□ Female

Q11. Educational level

□ High school

□ Bachelor’s degree

□ Master’s degree

□ Doctoral degree

**Section D. Main questionnaire items**

For the following sections, please indicate your agreement with each statement using a 5-point Likert scale.

1 = Strongly disagree; 2 = Disagree; 3 = Neutral; 4 = Agree; 5 = Strongly agree.

**Emotional Resonance (ER)**

| **Item** | **Statement** | **1** | **2** | **3** | **4** | **5** |
| --- | --- | --- | --- | --- | --- | --- |
| ER1 | I feel a strong emotional connection to the music in this metaverse music experience. | □ | □ | □ | □ | □ |
| ER2 | The emotions conveyed by the music match how I feel during this experience. | □ | □ | □ | □ | □ |
| ER3 | The music in this experience moves me emotionally. | □ | □ | □ | □ | □ |

**Aesthetic Novelty (AN)**

| **Item** | **Statement** | **1** | **2** | **3** | **4** | **5** |
| --- | --- | --- | --- | --- | --- | --- |
| AN1 | This metaverse music experience is unique in its look and sound. | □ | □ | □ | □ | □ |
| AN2 | The aesthetic design of this experience satisfies my curiosity. | □ | □ | □ | □ | □ |
| AN3 | The visual and auditory styling makes me feel like an adventurer. | □ | □ | □ | □ | □ |

**Social Presence (SP)**

| **Item** | **Statement** | **1** | **2** | **3** | **4** | **5** |
| --- | --- | --- | --- | --- | --- | --- |
| SP1 | I felt like I was in the presence of another person in this metaverse music experience. | □ | □ | □ | □ | □ |
| SP2 | I felt that the people in this metaverse music experience were aware of my presence. | □ | □ | □ | □ | □ |
| SP3 | I had a sense that I was interacting with other people in this experience, not just with the system. | □ | □ | □ | □ | □ |

**Social Experience (SE)**

| **Item** | **Statement** | **1** | **2** | **3** | **4** | **5** |
| --- | --- | --- | --- | --- | --- | --- |
| SE1 | I felt connected with artists during this metaverse music experience. | □ | □ | □ | □ | □ |
| SE2 | I felt a sense of togetherness with other audience members during this metaverse music experience. | □ | □ | □ | □ | □ |
| SE3 | I felt that the metaverse music experience was shared with others. | □ | □ | □ | □ | □ |

**Avatar Customization (AC)**

| **Item** | **Statement** | **1** | **2** | **3** | **4** | **5** |
| --- | --- | --- | --- | --- | --- | --- |
| AC1 | I can freely customize my avatar’s appearance in this metaverse music platform. | □ | □ | □ | □ | □ |
| AC2 | This metaverse music platform provides sufficient options to customize my avatar to my preferences. | □ | □ | □ | □ | □ |
| AC3 | I feel I have control over many aspects of my avatar’s look (e.g., clothing, accessories) in this metaverse music platform. | □ | □ | □ | □ | □ |

**Narrativity (NA)**

| **Item** | **Statement** | **1** | **2** | **3** | **4** | **5** |
| --- | --- | --- | --- | --- | --- | --- |
| NA1 | During this metaverse music experience, I was impatient to find out how the story would end. | □ | □ | □ | □ | □ |
| NA2 | During this metaverse music experience, I was eager to see how everything would play out. | □ | □ | □ | □ | □ |
| NA3 | This experience aroused my curiosity about what would happen next. | □ | □ | □ | □ | □ |

**Multi-sensory (MS)**

| **Item** | **Statement** | **1** | **2** | **3** | **4** | **5** |
| --- | --- | --- | --- | --- | --- | --- |
| MS1 | This experience makes a strong impression on my visual and other senses. | □ | □ | □ | □ | □ |
| MS2 | This metaverse music experience appeals to my senses. | □ | □ | □ | □ | □ |
| MS3 | I find this metaverse music experience interesting for discovering new sensory experiences. | □ | □ | □ | □ | □ |

**Flow Experience (FE)**

| **Item** | **Statement** | **1** | **2** | **3** | **4** | **5** |
| --- | --- | --- | --- | --- | --- | --- |
| FE1 | During this metaverse music experience, I was absorbed in what I was doing. | □ | □ | □ | □ | □ |
| FE2 | All my attention was on this metaverse music experience. | □ | □ | □ | □ | □ |
| FE3 | My actions flowed effortlessly during this metaverse music experience. | □ | □ | □ | □ | □ |

**Emotional Value Perception (EVP)**

| **Item** | **Statement** | **1** | **2** | **3** | **4** | **5** |
| --- | --- | --- | --- | --- | --- | --- |
| EVP1 | I enjoyed this metaverse music experience. | □ | □ | □ | □ | □ |
| EVP2 | I felt emotionally uplifted during this metaverse music experience. | □ | □ | □ | □ | □ |
| EVP3 | This metaverse music experience gave me pleasure. | □ | □ | □ | □ | □ |

**Technology Acceptance (TA)**

| **Item** | **Statement** | **1** | **2** | **3** | **4** | **5** |
| --- | --- | --- | --- | --- | --- | --- |
| TA1 | Learning to use this metaverse music platform is easy for me. | □ | □ | □ | □ | □ |
| TA2 | My interaction with this metaverse music platform is clear and understandable. | □ | □ | □ | □ | □ |
| TA3 | I find this platform uncomplicated and not mentally demanding to use. | □ | □ | □ | □ | □ |

**Content Creation Behavior (CCB)**

| **Item** | **Statement** | **1** | **2** | **3** | **4** | **5** |
| --- | --- | --- | --- | --- | --- | --- |
| CCB1 | I post logs/articles on metaverse music platforms. | □ | □ | □ | □ | □ |
| CCB2 | I post status updates on metaverse music platforms. | □ | □ | □ | □ | □ |
| CCB3 | I post photos/videos on metaverse music platforms. | □ | □ | □ | □ | □ |

**End of Survey**

Thank you for your participation!

Your responses have been recorded.
