# Supplementary material for: How metaverse music platform cues shape content creation behavior: Evidence from social and flow pathways
Source: PLoS One. 2026 May 4;21(5):e0348632. doi: 10.1371/journal.pone.0348632 (PMC13138650; doi:10.1371/journal.pone.0348632)
Supplement: S2 Appendix — (DOCX) [file pone.0348632.s002.docx]

**S2 Appendix**

**Measurement items**

Unless otherwise indicated, items were measured on a 5-point Likert scale (1 = strongly disagree; 5 = strongly agree).

**Emotional resonance (ER)**

Source: [38]

ER1. I feel a strong emotional connection to the music in this metaverse music experience.

ER2. The emotions conveyed by the music match how I feel during this experience.

ER3. The music in this experience moves me emotionally.

**Aesthetic novelty (AN)**

Source: [21]

AN1. This metaverse music experience is unique in its look and sound.

AN2. The aesthetic design of this experience satisfies my curiosity.

AN3. The visual and auditory styling makes me feel like an adventurer.

**Social presence (SP)**

Source: [23]

SP1. I felt like I was in the presence of another person in this metaverse music experience.

SP2. I felt that the people in this metaverse music experience were aware of my presence.

SP3. I had a sense that I was interacting with other people in this experience, not just with the system.

**Social experience (SE)**

Source: [39]

SE1. I felt connected with artists during this metaverse music experience.

SE2. I felt a sense of togetherness with other audience members during this metaverse music experience.

SE3. I felt that the metaverse music experience was shared with others.

**Avatar customization (AC)**

Source: [40]

AC1. I can freely customize my avatar’s appearance in this metaverse music platform.

AC2. This metaverse music platform provides sufficient options to customize my avatar to my preferences.

AC3. I feel I have control over many aspects of my avatar’s look (e.g., clothing, accessories) in this metaverse music platform.

**Narrativity (NA)**

Source: [41]

NA1. During this metaverse music experience, I was impatient to find out how the story would end.

NA2. During this metaverse music experience, I was eager to see how everything would play out.

NA3. This experience aroused my curiosity about what would happen next.

**Multi-sensory (MS)**

Source: [42]

MS1. This experience makes a strong impression on my visual and other senses.

MS2. This metaverse music experience appeals to my senses.

MS3. I find this metaverse music experience interesting for discovering new sensory experiences.

**Flow experience (FE)**

Source: [43]

FE1. During this metaverse music experience, I was absorbed in what I was doing.

FE2. All my attention was on this metaverse music experience.

FE3. My actions flowed effortlessly during this metaverse music experience.

**Emotional value perception (EVP)**

Source: [10]

EVP1. I enjoyed this metaverse music experience.

EVP2. I felt emotionally uplifted during this metaverse music experience.

EVP3. This metaverse music experience gave me pleasure.

**Technology acceptance (TA)**

Source: [34]

TA1. Learning to use this metaverse music platform is easy for me.

TA2. My interaction with this metaverse music platform is clear and understandable.

TA3. I find this platform uncomplicated and not mentally demanding to use.

**Content Creation Behavior (CCB)**

Source: [33]

CCB1. I post logs/articles on metaverse music platforms.

CCB2. I post status updates on metaverse music platforms.

CCB3. I post photos/videos on metaverse music platforms.
